# Therapeutic drug monitoring plays an important role in patients with noninfectious uveitis receiving adalimumab

**DOI:** 10.3389/fimmu.2025.1733644

**Published:** 2025-12-11

**Authors:** Weidong Cheng, Jiayu Xiao, Shengjie Li, Huaru Wang, Shasha Wang, Yujing Qian, Chan Zhao, Fei Gao, Xuzhen Qin, Meifen Zhang

**Affiliations:** 1 Department of Ophthalmology, Peking Union Medical College Hospital, Chinese Academy of Medical Sciences and Peking Union Medical College, Beijing, China; 2 Department of Laboratory Medicine, Peking Union Medical College Hospital, Chinese Academy of Medical Sciences and Peking Union Medical College, Beijing, China; 3 Institute of Clinical Medicine, National Infrastructure for Translational Medicine, State Key Laboratory of Complex Severe and Rare Diseases, Chinese Academy of Medical Sciences and Peking Union Medical College, Beijing, China

**Keywords:** drug monitoring, noninfectious uveitis, adalimumab, antidrug antibodies, tumor necrosis factor α

## Abstract

**Objective:**

To evaluate the association between serum adalimumab (ADA) concentrations, antibodies against adalimumab (AAAs), tumor necrosis factor α (TNFα) levels, and clinical response in patients with noninfectious uveitis (NIU), as well as changes in TNFα after administration of ADA, to explore the role of therapeutic drug monitoring in these patients.

**Methods:**

This retrospective study included NIU patients treated with ADA at Peking Union Medical College Hospital between June 2024 and April 2025, who underwent testing for serum ADA and AAA levels. Most patients also had serum TNFα measured concurrently. Clinical data collected included concomitant medications, ADA dosing frequency, and ocular inflammation status, etc. High-performance size-exclusion chromatography was used to characterize forms of TNFα in serum samples of patients. THP-1 cells were stimulated with free TNFα or TNFα-ADA complexes to compare their pro-inflammatory activity.

**Results:**

Among 164 test results from 147 patients included (aged 5~56 years), median ADA level was significantly lower in AAA-positive patients than in AAA-negative patients (1.9 vs. 6.4 μg/mL; P<0.001), and lower in those with active inflammation than in those with quiescent inflammation (2.2 vs. 6.0 μg/mL; P<0.001). An ADA level below 4.1 μg/mL was associated with poor clinical response. Concomitant antimetabolite use was linked to a lower proportion of detectable AAAs compared with ADA monotherapy (34.3% vs. 54.5%; P = 0.036). Median ADA level was significantly higher when testing occurred ≤14 days after the last dose of ADA (P<0.001), though many patients maintained therapeutic levels even with extended dosing intervals. TNFα levels increased in most patients after ADA therapy, predominantly in the form of TNFα-ADA complexes, which exhibited significantly weaker pro-inflammatory effects than free TNFα.

**Conclusion:**

The presence of AAAs was associated with reduced ADA levels and an increased risk of treatment failure. Despite the limitations of a retrospective design, these findings suggest that therapeutic drug monitoring may help identify causes of treatment failure and optimize regimens in stable patients.

## 1 Introduction

Noninfectious uveitis (NIU) is a group of autoimmune inflammatory ocular diseases, which may be seriously sight-threatening. Traditional therapeutic agents include topical and systemic corticosteroids, antimetabolites, calcineurin inhibitors, etc. Adalimumab (ADA), a fully humanized tumor necrosis factor α (TNFα) inhibitor, has been shown to be effective and safe in treating NIU in adults and adolescents (1–4). It was the first biological agent that was permitted by United States Food and Drug Administration to treat NIU. It is administered by subcutaneous injection, every two weeks initially. It’s typically used in cases refractory to conventional therapy, or as a first-line treatment for certain etiologies such as Behçet disease (5). However, some patients yet experienced treatment failure. And for those who had satisfactory response, there is currently no consensus on when the dosing interval can be prolonged and when it can be withdrawn. Several studies have reported the development of antibodies against ADA (AAAs) in patients with NIU, which can result in loss of medication efficacy of ADA (6–10). However, the conclusions of these studies are constrained by the limited sample size, and no studies have focused on the changes of serum TNFα levels in patients. The aim of this study was to investigate the relationship among circulating ADA levels, AAAs, TNFα and clinical response in Chinese patients receiving ADA therapy for NIU.

## 2 Materials and methods

### 2.1 Study design and patient recruitment

This single-site, retrospective cross-sectional study was conducted at the Department of Ophthalmology, Peking Union Medical College Hospital (PUMCH). NIU patients treated with ADA who underwent therapeutic drug monitoring (TDM), including measurements of serum ADA and AAA levels, from June 2024 to April 2025, were included. This study was approved by the PUMCH Ethics Review Committee, and due to its retrospective nature, informed consent was waived.

To minimize variability in ADA levels due to timing of drug administration, only patients who received TDM over 7 days after a scheduled dose of ADA were included, since it takes about 5 to 7 days for ADA to reach peak concentration.

### 2.2 Data collection

Demographic data including gender and age were obtained by medical record review. Diagnosis, duration of therapy, frequency of ADA administration, coexisting local and systemic treatment, ocular inflammatory activity status, serum ADA, AAA and TNFα level at the time of laboratory examination were documented.

ADA and AAA levels were tested in serum samples using reagents produced by Changde Horui Biotechnology Ltd, China, and analyzers produced by Suzhou Helmen Precision Instruments Ltd, China, based on the principle of fluorescence immunochromatography assay. And TNFα levels were measured using reagents and analyzers produced by Siemens Healthcare Diagnostics Products Ltd, UK, based on the principle of chemiluminescent immunometric assay. The lower and upper limits of quantification for ADA levels are <0.4 μg/mL and >500 μg/mL, respectively; for AAA levels, they are <4 ng/mL and >500 ng/mL, respectively. And for TNFα, the analytical sensitivity is 1.7 pg/mL.

Clinical response was categorized as active or inactive inflammation. Inactive inflammation was determined if the following criteria were all met in both eyes: grade 0 of anterior chamber cells and vitreous haze; resolution or quiescence of previous inflammatory choroidal or retinal lesions; no appearance of new inflammatory choroidal or retinal lesions; and no newly added topical or systemic anti-inflammatory medications at the last visit.

### 2.3 Characterization of TNFα forms

A subset of serum samples from included patients were fractionated by high-performance size-exclusion chromatography (HP-SEC) to characterize ex vivo forms of TNFα. Serum was diluted 1:1 in PBS and filtered (0.22 μm filter) before applying 1.0 mL to a Superdex 200 10/300 GL column (GE Healthcare, UK) and eluted with PBS (0.75 mL/min). Elution profiles were monitored by measuring absorption at 280 nm with an ÄKTA explorer high-performance liquid chromatography system (GE Healthcare, UK). Samples spiked with free TNFα (stored in 6% human serum albumin) [TNFα (500 pg/mL) supplemented with IVIg (5 mg/mL)] and TNFα-ADA complexes [TNFα (500 pg/mL) and ADA (5 μg/mL), supplemented with IVIg (5 mg/mL)] were used as controls (11). Fractions of 0.5 mL were collected and TNFα concentrations were measured using reagents and analyzers mentioned above.

### 2.4 Comparison of pro-inflammatory activity between free TNFα and TNFα-ADA complexes

Take P3 generation of THP-1 cells (EallBio Life Sciences, China) and inoculate them in 96-well plates at a density of 1×10^5^ cells per well. Add complete culture medium containing 1, 5, 25, and 125 ng/mL free TNFα and TNFα-ADA complexes respectively. Set 0 ng/mL as the blank control. Each group has 6 duplicate wells. Place them in a CO_2_ incubator for 48 hours. Then, collect the supernatant of the culture medium and use the cytokine multiplex detection kits (Cellgene Biotechnology, China) to detect the levels of pro-inflammatory cytokines such as interleukin (IL) -1β, IL-6, IL-8 and interferon γ (IFNγ) in the supernatant.

### 2.5 Statistical analysis

Statistical analyses were performed using MedCalc, version 23.2.1 (MedCalc Software Ltd, Belgium). Data were expressed as median for continuous variables and frequency (percentage) for qualitative variables. Comparisons of continuous variables were performed using the Mann-Whitney *U* test. χ^2^ test was used for comparisons involving qualitative variables. A binary logistic regression model was used to generate receiver operating characteristic (ROC) curves to determine threshold ADA level associated with inflammatory activity. Statistical significance was defined as P<0.05.

## 3 Results

A total of 147 patients with NIU receiving ADA therapy were included, each of whom underwent TDM at least once. The median (IQR) age was 14.0 (11.0~22.0) years (range, 5.0~56.0 years), and 64.6% were adolescents under 18 years old. 73 patients (49.7%) were female and 74 were male (50.3%). The median (IQR) time from ADA therapy initiation to laboratory examination was 18.0 (9.0~34.5) months (range, 3.0~122.0 months). Bilateral disease was present in 121 patients (82.3%). Specific disease etiologies among included patients are listed in **Table 1**.

**Table 1:** Diagnoses of included patients.

Diagnosis		No. (%)
Behçet disease		18 (12.2)
Blau syndrome		2 (1.4)
HLA-B27-associated uveitis		2 (1.4)
Idiopathic uveitis	Anterior	4 (2.7)
	Anterior and intermediate	45 (30.6)
	Posterior	8 (5.4)
	Panuveitis	41 (27.9)
Juvenile idiopathic arthritis-associated uveitis		20 (13.6)
Posner-Schlossman syndrome		1 (0.7)
Scleritis		2 (1.4)
Vogt-Koyanagi-Harada disease		4 (2.7)

A total of 164 TDM results were included in the analysis. The median (IQR) ADA level was 5.3 (2.1~8.2) μg/mL. AAAs were present in 62 tests (37.8%). Serum TNFα levels were tested at the same time of 144 examinations, and the median (IQR) level was 140.5 (31.0~247.5) pg/mL.

### 3.1 Association between ADA levels and presence of AAAs

Notably, median ADA level was lower in patients with AAAs than in those without AAAs (1.9 vs. 6.4 μg/mL; P<0.001). When stratified by clinical response, with or without concomitant immunomodulatory drugs and dosing intervals of ADA, median ADA level remained lower in AAA-positive patients, compared with AAA-negative patients (**Table 2**).

**Table 2:** ADA levels based on presence or absence of AAAs, stratified by different perspectives.

Stratifications	No. (%)	ADA level, median, μg/mL		P value
		AAA present	AAA absent	
Active inflammation	45 (27.4)	<0.4	4.9	<0.001
Inactive inflammation	119 (72.6)	4.2	6.9	<0.001
Without concomitant drugs	44 (26.8)	1.5	6.9	0.005
With concomitant drugs	120 (73.2)	2.2	6.3	<0.001
Every 2 weeks	75 (45.7)	4.0	8.8	<0.001
Less frequent dosing	89 (54.3)	0.7	4.5	<0.001

### 3.2 Association between clinical response and ADA levels or presence of AAAs

To investigate the relationship among clinical response, ADA level and presence of AAAs, the cohort was stratified based on active or inactive ocular inflammation. A total of 45 examinations (27.4%) were performed while the inflammation was active. The median ADA level was 2.2 μg/mL in patients with active inflammation, which was significantly lower than 6.0 μg/mL in patients with quiescent inflammation (P<0.001). Furthermore, the proportion of patients with detectable AAAs was higher in the active group (23 of 45 [51.1%]) compared with the inactive group (39 of 119 [32.8%]; P = 0.031). The trend was similar when stratified by coexisting drugs and dosing frequency of ADA, but there was no significant difference in some situations (**Table 3**).

**Table 3:** ADA levels and proportion of AAA present in active or inactive ocular inflammatory status, stratified by different perspectives.

Stratifications	ADA level, median, μg/mL		P value
	Active inflammation	Inactive inflammation	
Total	2.2	6.0	<0.001
Without concomitant drugs	2.1	4.5	0.221
With concomitant drugs	3.4	6.2	<0.001
Every 2 weeks	3.8	7.9	0.009
Less frequent dosing	1.7	4.5	0.002

Stratifications	AAA present, %		P value
	Active inflammation	Inactive inflammation	
Total	51.1	32.8	0.031
Without concomitant drugs	63.6	51.5	0.489
With concomitant drugs	47.1	25.6	0.023
Every 2 weeks	50.0	29.1	0.095
Less frequent dosing	52.0	35.9	0.168

**Figure 1:**
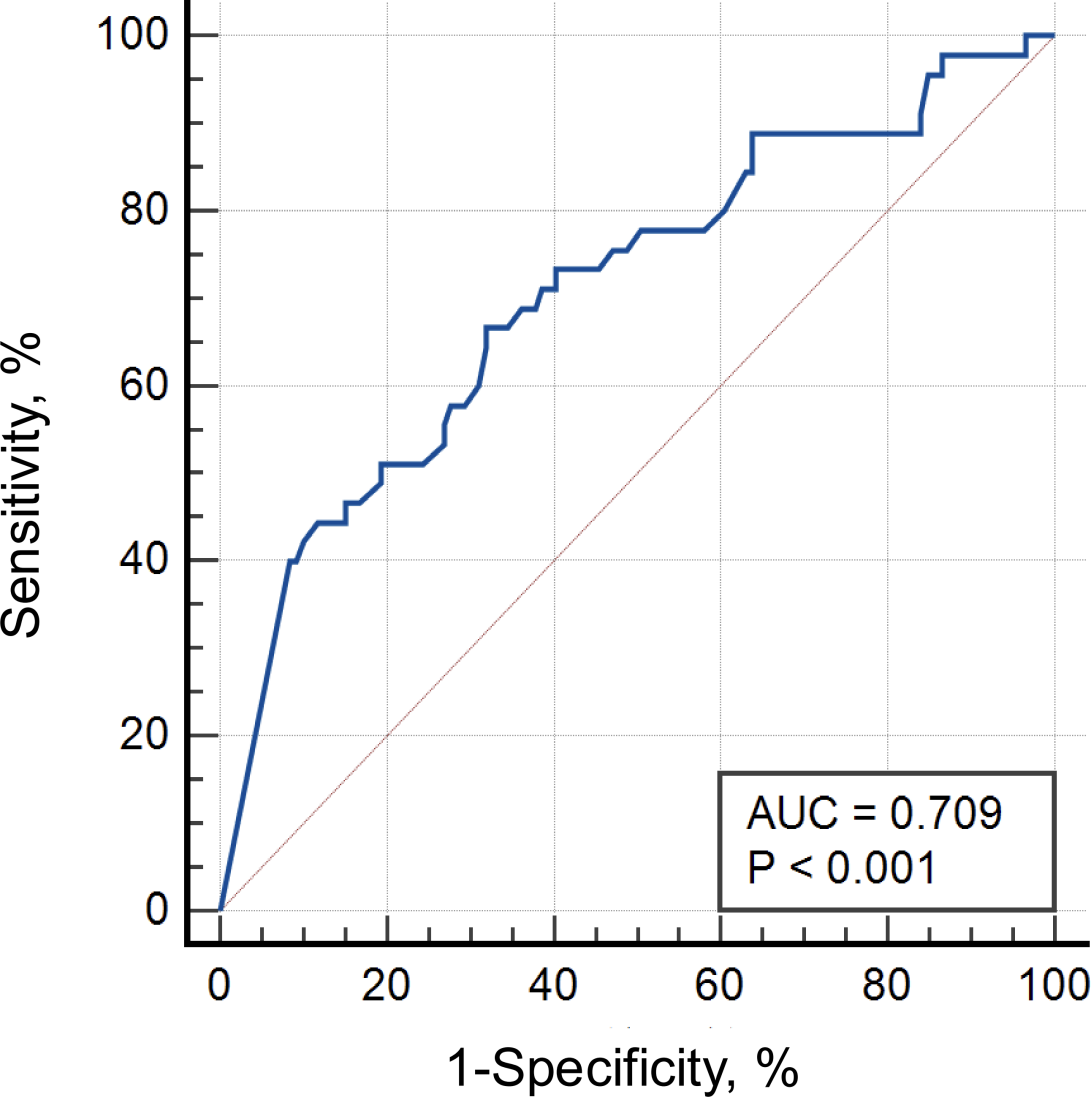
Receiver operating characteristic curve of ADA levels for clinical response.

Analysis of ROC curve demonstrated that an ADA level below the threshold of 4.1 μg/mL was associated with active inflammation, with a sensitivity of 66.7% (95% CI, 51.0%~80.0%) and specificity of 68.1% (95% CI, 58.9%~76.3%) and an area under the curve (AUC) of 0.709, P<0.001 (**Figure 1**).

### 3.3 Influence of concomitant immunosuppressors on presence of AAAs

The influence of concomitant immunosuppressors on ADA levels and production of AAAs was analyzed. 44 tests (26.8%) were performed when patients were using ADA as systemic monotherapy, while others were performed when patients were taking at least one kind of other immunomodulatory drugs, such as low-dose oral corticosteroids, antimetabolites, calcineurin inhibitors, etc. There was no significant difference in median ADA level between patients with or without concomitant drugs (5.7 vs. 4.0 μg/mL; P = 0.065). But when it comes to the presence of AAAs, the proportion was lower in the antimetabolite (including methotrexate, mycophenolate mofetil and azathioprine) group compared with the ADA monotherapy group (34.3% vs. 54.5%; P = 0.036). On the other hand, calcineurin inhibitors (including cyclosporine and tacrolimus) did not reduce the proportion of AAA positivity (**Table 4**).

**Table 4:** Proportion of AAA present, stratified by different concomitant systemic immunomodulatory drugs.

AAA present, %				P value
Without concomitant drugs	54.5	With concomitant drugs	31.7	0.008
		With one kind of antimetabolites	34.3	0.036
		With one kind of calcineurin inhibitors	54.5	1.000

### 3.4 Association between dosing time and ADA levels

**Figure 2:**
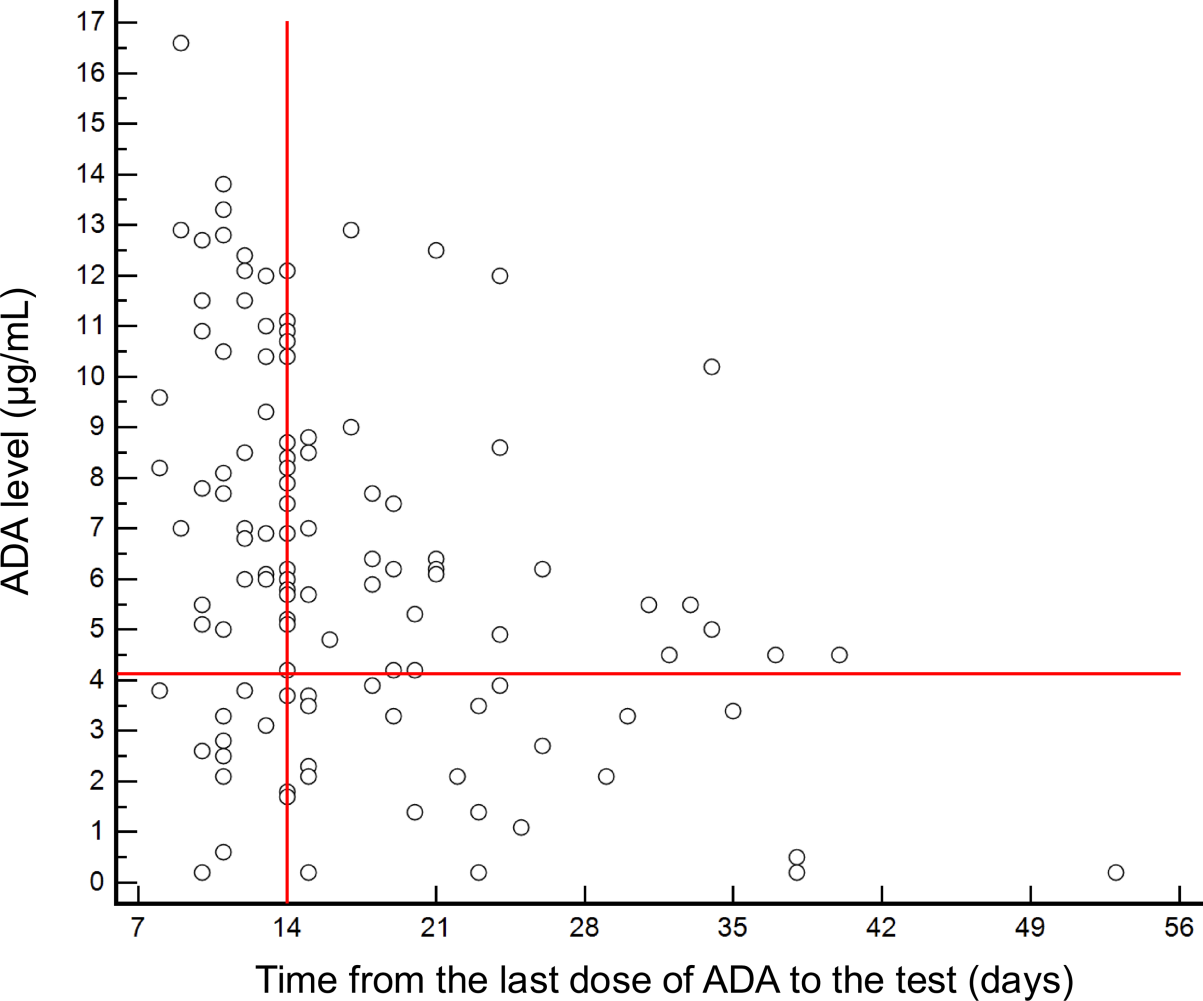
Relationship between ADA levels and time interval since last dose of ADA.

We subsequently investigated the relationship between ADA levels and the time interval since the last administration of ADA. To eliminate the influence of AAAs on ADA levels, we only retained the test results without detectable AAAs and generated a scatter plot (**Figure 2**). There was significant inter-individual variability in pharmacokinetics of ADA. The median ADA level was 7.0 μg/mL for intervals of ≤14 days, which was significantly higher than the median ADA level of 4.5 μg/mL for longer intervals (P<0.001). However, a considerable proportion of patients with extended dosing intervals still maintained ADA levels above the effective threshold of 4.1 μg/mL, and 90.6% of these patients were under stable control of ocular inflammation.

### 3.5 Changes of TNFα levels after ADA therapy

The correlation among serum TNFα levels, presence of AAAs and clinical response was explored. Analysis demonstrated that median TNFα level was significantly higher in absence of AAAs, and in patients whose ocular inflammation was quiescent (**Table 5**).

**Table 5:** TNFα level stratified by presence or absence of AAAs and clinical response.

Stratifications	No. (%)	TNFα level, median (IQR), pg/mL	P value
AAA present	57 (39.6)	65.5 (15.0~212.0)	<0.001
AAA absent	87 (60.4)	174.0 (113.3~261.5)	
Active inflammation	35 (24.3)	53.9 (15.7~172.8)	0.002
Inactive inflammation	109 (75.7)	159.0 (90.0~257.8)	

**Figure 3:**
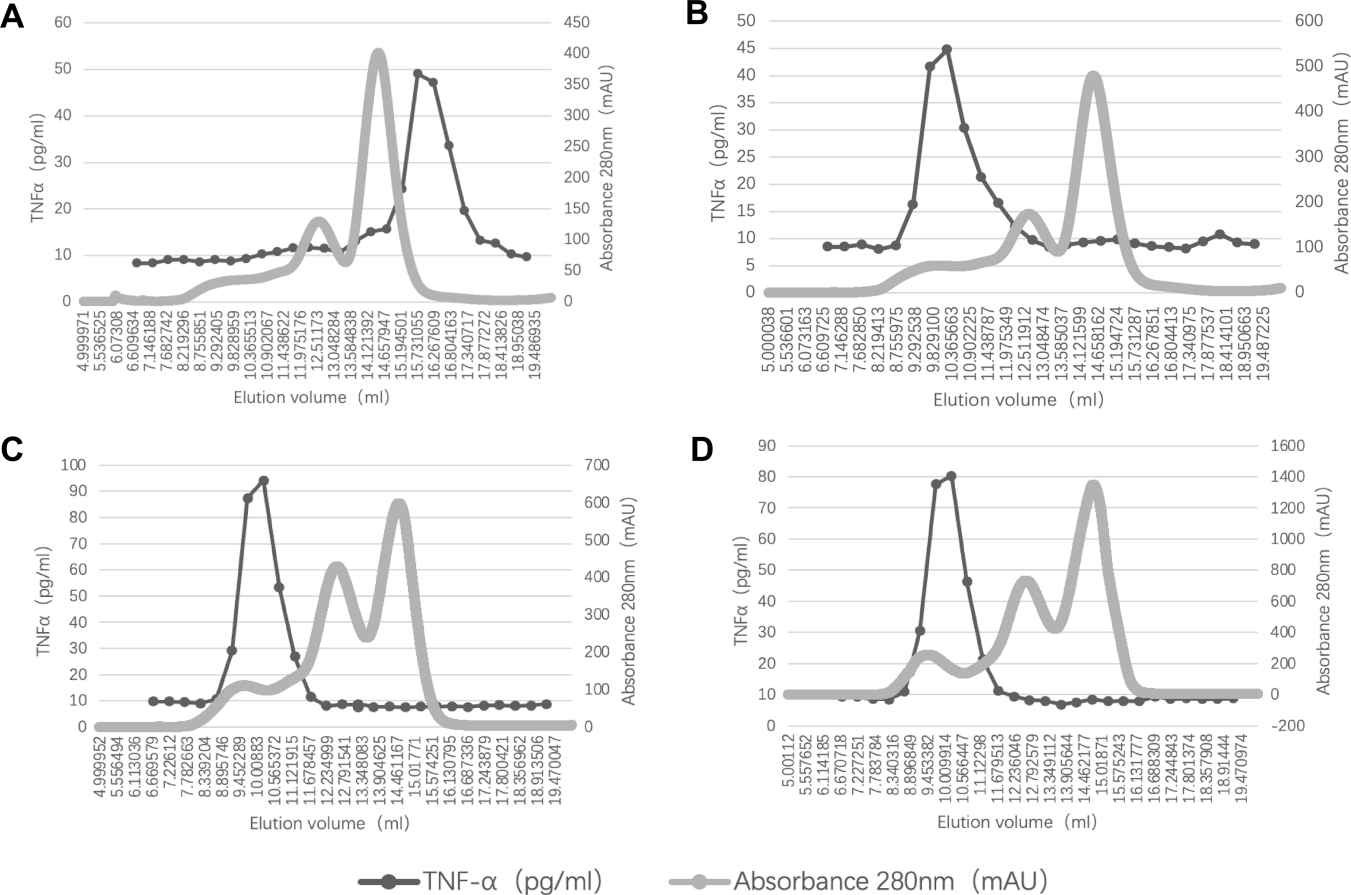
Characterization of forms of TNFα. **(A)** Control sample of free TNFα; **(B)** Control sample of TNFα-ADA complexes; **(C, D)** Serum samples of NIU patients after ADA therapy.

An interesting phenomenon emerged. We traced back 43 results of TNFα level before administration of ADA. It turned out that TNFα levels increased in 42 of 43 patients (97.7%) after ADA therapy. To characterize the forms of TNFα, we analyzed several serum samples of included patients with HP-SEC. We first fractionated standard control samples of free TNFα and TNFα-ADA complexes. TNFα levels were measured in collected fractions. Free TNFα was eluted in the later fractions since the molecular volume was small (**Figure 3A**), whereas for TNFα-ADA complexes, the TNFα peak shifted to the left (**Figure 3B**). Next, we characterized serum samples (n=6) with the same protocol and compared the location of TNFα peak with control samples. Two representative graphs of patients are shown (**Figures 3C, D**). The TNFα peaks of patients’ sera consistently overlapped with control samples of TNFα-ADA complexes, which means after the use of ADA, the majority forms of elevated TNFα were TNFα-ADA complexes.

Subsequently, we compared the differences in pro-inflammatory activity between free TNFα and TNFα-ADA complexes. It was clear that THP-1 cells secreted significantly higher levels of IL-1β, IL-6, IL-8 and IFNγ when they were stimulated with free TNFα, compared with TNFα-ADA complexes (**Table 6**). It demonstrated that the pro-inflammatory activity of TNFα-ADA complexes was significantly lower than free TNFα.

**Table 6:** Levels of pro-inflammatory cytokines after THP-1 cells were treated with free TNFα and TNFα-ADA complexes.

Groups	Cytokine levels, median, pg/ml							
	IL-1β	P value	IL-6	P value	IL-8	P value	IFNγ	P value
TNFα 1ng/mL	1.98	0.048	3.03	0.011	176.24	0.012	0.38	0.562
TNFα 1ng/mL +ADA	1.21		1.39		51.79		0.32	
TNFα 5ng/mL	4.30	<0.001	7.59	<0.001	550.40	<0.001	1.04	0.001
TNFα 5ng/mL +ADA	1.68		2.22		115.97		0.49	
TNFα 25ng/mL	11.77	0.003	19.79	<0.001	1315.87	<0.001	2.84	<0.001
TNFα 25ng/mL +ADA	4.75		6.18		299.62		0.61	
TNFα 125ng/mL	22.58	0.001	40.09	<0.001	2651.78	<0.001	3.13	<0.001
TNFα 125ng/mL +ADA	9.79		16.32		799.58		0.77	

## 4 Discussion

This study provides one of the largest sample sizes in analyzing the role of TDM with ADA therapy in patients with NIU. Our findings demonstrated that the presence of AAAs was associated with lower ADA levels and risk of treatment failure. ROC analysis found that ADA level above 4.1 μg/mL was associated with favorable clinical response. Although concomitant immunosuppressants did not influence ADA levels, we found that antimetabolites could significantly reduce the production of AAAs; however, calcineurin inhibitors could not. Patients whose time intervals from last dose of ADA to TDM were ≤14 days had a significantly higher median ADA level than patients with longer intervals, but there were many patients with prolonged dosing intervals who could also keep ADA levels above the effective threshold. We unexpectedly found that in the vast majority of patients, serum TNFα levels had risen after administration of ADA, and higher TNFα levels were associated with the absence of AAAs and inactive ocular inflammation. Furthermore, we revealed that the majority forms of elevated TNFα were TNFα-ADA complexes. This result was observed for the first time in patients with NIU.

The immunogenicity of ADA, manifested as formation of AAAs, is an important therapeutic consideration when prescribing ADA for patients with NIU. A sustained antidrug response to biological agents depends on the formation of IgG antibodies that target the antigen binding sites of monoclonal antibodies and are thought to be neutralizing (12). AAAs are reported to develop as early as 2 weeks or up to several years after administration of ADA in rheumatological diseases (13).

Brunelli et al. (14) reported that AAAs reached a peak frequency of 37% at 6 months in patients with juvenile idiopathic arthritis after ADA initiation. In our study, AAAs were detectable in 37.8% of tests, which was in line with limited data on NIU, ranged from 2.7% and 5% in the VISUAL I (1) and VISUAL II (2) trials, respectively, to 13.6% and 45% in smaller prospective studies and retrospective case series (6–9). It was reported in previous studies that risk factors for AAA formation included higher disease activity, longer disease duration, concomitant infection, intramuscular route of administration, and specific human leukocyte antigen alleles such as HLA-DQA1*05 (12–17).

Regardless of inflammation activity, concomitant therapy and dosing interval, the presence of AAAs was associated with lower ADA levels in this cohort. When clinical response was compared, the general trend was that low ADA levels and presence of AAAs were more likely to lead to treatment failure. However, in some stratified analyses, the difference wasn’t statistically significant, especially in ADA monotherapy group, which was due to sample size perhaps.

Concurrent antimetabolite use, particularly methotrexate, had been reported to be associated with lower rates of AAA formation in patients with systemic rheumatologic diseases (13, 17–19). However, evidence supporting that antimetabolites could reduce AAAs in patients with NIU is limited. Skrabl-Baumgartner et al. (7) reported that patients with juvenile idiopathic arthritis-associated uveitis treated with ADA who did not form AAAs were more likely to be receiving a concurrent antimetabolite; on the contrary, Pichi et al (10) did not find a significant influence of antimetabolites on AAA formation. Besides, Bellur et al (9) found that mycophenolate mofetil could reduce AAA levels, whereas methotrexate could not. Consistent with some prior reports, our study found that concomitant antimetabolite use was associated with a lower likelihood of AAA detection. Nevertheless, only one kind of calcineurin inhibitors did not reduce AAA formation, which was discovered for the first time.

Several studies showed that TDM in ADA-treated NIU patients may be beneficial. Sejournet et al (8) and Pichi et al (10) reported that AAA formation, low ADA levels together with therapy failure brought about increased ADA dosing frequency, an increased dose and intraclass or interclass treatment switching, suggesting that TDM may be useful in determining treatment adjustment. Additionally, TDM in rheumatologic and inflammatory bowel diseases has been associated with reduced treatment costs and facilitated treatment optimization (20–23). In our study, we found that a substantial proportion of patients on extended dosing intervals maintained ADA levels above the identified therapeutic threshold. It indicated that TDM could help minimize overexposure of ADA.

While ADA is one kind of TNFα inhibitors, it seemed strange that most patients’ TNFα level rose up after the administration of ADA. Our experiments on representative serum samples of patients demonstrated that the dominant forms of TNFα after ADA therapy were TNFα-ADA complexes, whose pro-inflammatory effect was significantly weaker than free TNFα. Conversely, low serum TNFα levels reflect that ADA may combine with AAAs preferentially rather than free TNFα, since free TNFα is rapidly cleared from the circulation, but TNFα-ADA complex has a prolonged half-life (11).

There are some limitations in our study. The retrospective nature of the study meant that there were variable dosing intervals of ADA, and some examinations were not performed exactly on the day of trough drug concentrations, limiting the representativeness of pharmacokinetics. Despite these limitations, this study provides one of the largest sample sizes focusing on the role of TDM of ADA in patients with NIU.

## 5 Conclusion

This study underscores the importance of TDM during ADA therapy in NIU. Patients with sufficiently high AAA levels may have negligible ADA concentrations, leading to loss of effectiveness. For these patients, it is necessary to consider therapy switching to another TNFα inhibitor or a different type of immunomodulatory drugs. Furthermore, less frequent dosing may maintain serum ADA concentrations above effective level in a subset of patients. TDM could be beneficial in reducing overexposure of ADA in these patients. Further prospective studies are needed to clarify the role of TDM in ADA treatment in patients with NIU.

## Data Availability

The raw data supporting the conclusions of this article will be made available by the authors, without undue reservation.

## References

[1] Jaffe GJ Dick AD Brezin AP Nguyen QD Thorne JE Kestelyn P . Adalimumab in patients with active noninfectious uveitis. N Engl J Med. (2016) 375:932–43. doi: 10.1056/NEJMoa1509852, PMID: 27602665

[2] Nguyen QD Merrill PT Jaffe GJ Dick AD Kurup SK Sheppard J . Adalimumab for prevention of uveitic flare in patients with inactive non-infectious uveitis controlled by corticosteroids (Visual ii): A multicentre, double-masked, randomised, placebo-controlled phase 3 trial. Lancet. (2016) 388:1183–92. doi: 10.1016/S0140-6736(16)31339-3, PMID: 27542302

[3] Ramanan AV Dick AD Jones AP McKay A Williamson PR Compeyrot-Lacassagne S . Adalimumab plus methotrexate for uveitis in juvenile idiopathic arthritis. N Engl J Med. (2017) 376:1637–46. doi: 10.1056/NEJMoa1614160, PMID: 28445659

[4] Quartier P Baptiste A Despert V Allain-Launay E Kone-Paut I Belot A . Adjuvite: A double-blind, randomised, placebo-controlled trial of adalimumab in early onset, chronic, juvenile idiopathic arthritis-associated anterior uveitis. Ann Rheum Dis. (2018) 77:1003–11. doi: 10.1136/annrheumdis-2017-212089, PMID: 29275333

[5] Hatemi G Christensen R Bang D Bodaghi B Celik AF Fortune F . 2018 Update of the eular recommendations for the management of Behcet’s syndrome. Ann Rheum Dis. (2018) 77:808–18. doi: 10.1136/annrheumdis-2018-213225, PMID: 29625968

[6] Cordero-Coma M Calleja-Antolin S Garzo-Garcia I Nunez-Garnes AM Alvarez-Castro C Franco-Benito M . Adalimumab for treatment of noninfectious uveitis: immunogenicity and clinical relevance of measuring serum drug levels and antidrug antibodies. Ophthalmology. (2016) 123:2618–25. doi: 10.1016/j.ophtha.2016.08.025, PMID: 27692527

[7] Skrabl-Baumgartner A Seidel G Langner-Wegscheider B Schlagenhauf A Jahnel J . Drug monitoring in long-term treatment with adalimumab for juvenile idiopathic arthritis-associated uveitis. Arch Dis Child. (2019) 104:246–50. doi: 10.1136/archdischild-2018-315060, PMID: 30026253

[8] Sejournet L Kerever S Mathis T Kodjikian L Jamilloux Y Seve P . Therapeutic drug monitoring guides the management of patients with chronic non-infectious uveitis treated with adalimumab: A retrospective study. Br J Ophthalmol. (2022) 106:1380–6. doi: 10.1136/bjophthalmol-2021-319072, PMID: 33875451

[9] Bellur S McHarg M Kongwattananon W Vitale S Sen HN Kodati S . Antidrug antibodies to tumor necrosis factor alpha inhibitors in patients with noninfectious uveitis. JAMA Ophthalmol. (2023) 141:150–6. doi: 10.1001/jamaophthalmol.2022.5584, PMID: 36547953 PMC9936342

[10] Pichi F Smith SD AlAli SH Neri P . Adalimumab drug monitoring and treatment adjustment to drug antibodies in noninfectious uveitis. Am J Ophthalmol. (2024) 268:306–11. doi: 10.1016/j.ajo.2024.09.008, PMID: 39271091

[11] Berkhout LC l’Ami MJ Ruwaard J Hart MH Heer PO Bloem K . Dynamics of circulating Tnf during adalimumab treatment using a drug-tolerant Tnf assay. Sci Transl Med. (2019) 11:eaat3356. doi: 10.1126/scitranslmed.aat3356, PMID: 30700574

[12] Atiqi S Hooijberg F Loeff FC Rispens T Wolbink GJ . Immunogenicity of Tnf-inhibitors. Front Immunol. (2020) 11:312. doi: 10.3389/fimmu.2020.00312, PMID: 32174918 PMC7055461

[13] Thomas SS Borazan N Barroso N Duan L Taroumian S Kretzmann B . Comparative immunogenicity of tnf inhibitors: impact on clinical efficacy and tolerability in the management of autoimmune diseases. A systematic review and meta-analysis. BioDrugs. (2015) 29:241–58. doi: 10.1007/s40259-015-0134-5, PMID: 26280210

[14] Brunelli JB Silva CA Pasoto SG Saa CGS Kozu KT Goldenstein-Schainberg C . Anti-adalimumab antibodies kinetics: an early guide for juvenile idiopathic arthritis (Jia) switching. Clin Rheumatol. (2020) 39:515–21. doi: 10.1007/s10067-019-04798-6, PMID: 31707543

[15] Atzeni F Talotta R Salaffi F Cassinotti A Varisco V Battellino M . Immunogenicity and autoimmunity during anti-Tnf therapy. Autoimmun Rev. (2013) 12:703–8. doi: 10.1016/j.autrev.2012.10.021, PMID: 23207283

[16] Sazonovs A Kennedy NA Moutsianas L Heap GA Rice DL Reppell M . Hla-dqa1*05 carriage associated with development of anti-drug antibodies to infliximab and adalimumab in patients with Crohn’s disease. Gastroenterology. (2020) 158:189–99. doi: 10.1053/j.gastro.2019.09.041, PMID: 31600487

[17] De Simone C Amerio P Amoruso G Bardazzi F Campanati A Conti A . Immunogenicity of anti-tnfalpha therapy in psoriasis: A clinical issue? Expert Opin Biol Ther. (2013) 13:1673–82. doi: 10.1517/14712598.2013.848194, PMID: 24107126

[18] Mc Gettigan N Afridi AS Harkin G Lardner C Patchett S Cheriyan D . The optimal management of anti-drug antibodies to infliximab and identification of anti-drug antibody values for clinical outcomes in patients with inflammatory bowel disease. Int J Colorectal Dis. (2021) 36:1231–41. doi: 10.1007/s00384-021-03855-4, PMID: 33515082

[19] Emi Aikawa N de Carvalho JF Artur Almeida Silva C Bonfa E . Immunogenicity of anti-Tnf-alpha agents in autoimmune diseases. Clin Rev Allergy Immunol. (2010) 38:82–9. doi: 10.1007/s12016-009-8140-3, PMID: 19565360

[20] Papamichael K Juncadella A Wong D Rakowsky S Sattler LA Campbell JP . Proactive therapeutic drug monitoring of adalimumab is associated with better long-term outcomes compared with standard of care in patients with inflammatory bowel disease. J Crohns Colitis. (2019) 13:976–81. doi: 10.1093/ecco-jcc/jjz018, PMID: 30689771 PMC6939875

[21] Pedersen L Szecsi PB Johansen PB Bjerrum PJ . Evaluation of therapeutic drug monitoring in the clinical management of patients with rheumatic diseases: data from a retrospective single-center cohort study. Biologics. (2020) 14:115–25. doi: 10.2147/btt.S262511, PMID: 33162753 PMC7643816

[22] Martelli L Olivera P Roblin X Attar A Peyrin-Biroulet L . Cost-effectiveness of drug monitoring of anti-Tnf therapy in inflammatory bowel disease and rheumatoid arthritis: A systematic review. J Gastroenterol. (2017) 52:19–25. doi: 10.1007/s00535-016-1266-1, PMID: 27665099

[23] Heron V Afif W . Update on therapeutic drug monitoring in Crohn’s disease. Gastroenterol Clin North Am. (2017) 46:645–59. doi: 10.1016/j.gtc.2017.05.014, PMID: 28838420

